# Advances in Synergistic Corrosion Mechanisms of and Management Strategies for Impurity Gases During Supercritical CO_2_ Pipeline Transportation

**DOI:** 10.3390/molecules30204094

**Published:** 2025-10-15

**Authors:** Yutong Yan, Weifeng Lyu, Hongwei Yu, Wenfeng Lv, Keqiang Wei, Lichan Jiang

**Affiliations:** 1 University of Chinese Academy of Sciences, Beijing 100049, China; yanyutong23@mails.ucas.ac.cn (Y.Y.);; 2 Institute of Porous Flow and Fluid Mechanics, Chinese Academy of Sciences, Langfang 065007, China; 3 State Key Laboratory of Enhanced Oil & Gas Recovery, Beijing 100083, China; 4 Research Institute of Petroleum Exploration and Development, Beijing 100083, China

**Keywords:** supercritical CO_2_ transport, impurity gas effects, synergistic corrosion mechanism, impurity concentration threshold, pipeline integrity

## Abstract

Supercritical CO_2_ (sCO_2_) pipeline transport is a critical link for the large-scale implementation of Carbon Capture, Utilization, and Storage (CCUS) technology, yet its safety is severely challenged by residual impurity gases (e.g., H_2_O, O_2_, SO_2_, H_2_S, and NO_2_) from the capture process. This review systematically consolidates recent research advances, with the key findings being the following. Firstly, it reveals that the nonlinear synergistic effects among impurities are the primary cause of uncontrolled corrosion, whose destructive impact far exceeds the simple sum of individual effects. Secondly, it delineates the specific roles and critical thresholds of different impurities within the corrosion chain reaction, providing a theoretical basis for targeted control. Consequently, engineering management must enforce strict impurity concentration thresholds integrated with material upgrades and dynamic operational optimization. Future research should focus on developing multi-impurity reaction kinetic models, elucidating long-term corrosion product layer evolution, and establishing standardized experimental systems. This review provides crucial theoretical support for establishing impurity control standards and optimizing anti-corrosion strategies for the safe transport of CO_2_ in supercritical CCUS pipelines.

## 1. Introduction

**Figure 1:**
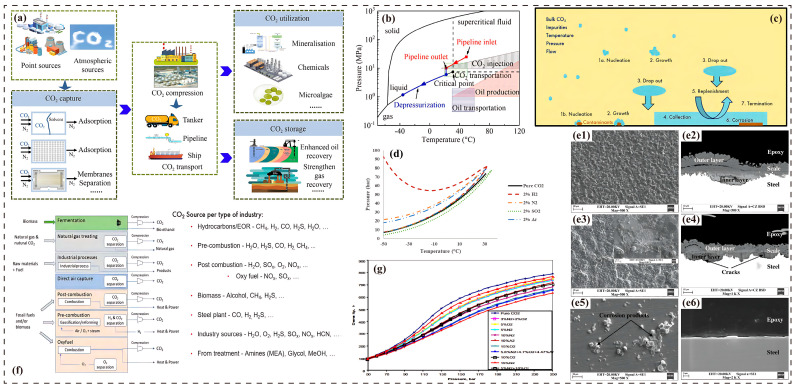
(**a**) Schematic diagram of the CCUS process [11]; (**b**) phase diagram of CO_2_ and various operations involved in Carbon Capture and Storage (CCS)/CCUS [12]; (**c**) CO_2_ corrosion process in the presence of H_2_O [13]; (**d**) density–pressure relationship curve of CO_2_ mixtures at 330 K [14]; (**e1**–**e6**) morphology and composition analysis of corrosion product scales formed on steel surfaces after 240 h of exposure in an sCO_2_ environment at 10 MPa, 80 °C, and a flow velocity of 1 m/s: (**e1**,**e2**) P110 steel, (**e3**,**e4**) 3Cr steel, and (**e5**,**e6**) 316L stainless steel [15]; (**f**) impurity gases in CO_2_ captured by different technologies [13]; (**g**) phase equilibrium of pure CO_2_ and CO_2_ with impurities [16].

Global warming exhibits a significant correlation with the rising atmospheric CO_2_ concentration. According to the Intergovernmental Panel on Climate Change (IPCC)’s Sixth Assessment Report, since the industrial revolution, the atmospheric CO_2_ concentration has increased from 280 ppm to 420 ppm, directly leading to a global surface temperature rise of approximately 1.1 °C [1]. To address this severe challenge, CCUS technology has emerged as a critical pathway for reducing the atmospheric CO_2_ concentration and achieving carbon neutrality goals. Among CCUS components, CO_2_ pipeline transport, as the core link connecting the capture end to the storage end, widely employs supercritical state transmission technology to balance transport efficiency with economic viability (Figure 1a,b) [2,3,4,5,6]. However, constrained by the current maturity and economic considerations of capture technologies, the captured CO_2_ stream inevitably contains impurity gases such as H_2_O, O_2_, SO_2_, and H_2_S. These impurities not only significantly alter the phase behavior of CO_2_, causing shifts in the critical point and expanding the gas–liquid two-phase region, but also induce severe corrosion issues (Figure 1d,f,g) [7,8,9,10].

The corrosion mechanism of CO_2_ and its impurity gases on tubular materials primarily manifests as an electrochemical corrosion process. In aqueous environments, CO_2_ dissolves to form carbonic acid (H_2_CO_3_), which further ionizes to produce H^+^, HCO_3_^−^, and CO_3_^2−^, constituting a corrosive medium. The predominant cathodic reaction is hydrogen ion reduction (2H^+^ + 2e^−^ → H_2_), while the anodic reaction is iron dissolution (Fe → Fe^2+^ + 2e^−^) (Figure 1c,e) [17]. The presence of impurity gases like H_2_S, SO_2_, and O_2_ significantly alters this corrosion process: HS generated from dissolved H_2_S forms a highly conductive FeS film with Fe^2+^, accelerating localized corrosion; SO_2_ dissolved in water forms sulfurous acid (H_2_SO_3_), further lowering the solution pH and promoting Fe dissolution; and O_2_ participates in the cathodic oxygen reduction reaction (O_2_ + 2H_2_O + 4e^−^ → 4OH^−^), increasing the corrosion rate [18,19,20,21]. These impurity gases not only change the corrosion reaction pathways but also influence the overall corrosion behavior of the material by affecting the composition and structure of the corrosion product film [22]. More critically, impurities can alter the phase equilibrium of the CO_2_–H_2_O system, lowering the solubility threshold of water. This leads to an increased likelihood of free water phase precipitation in impurity-containing systems under the same water content, thereby triggering electrochemical corrosion [23,24]. Therefore, in-depth investigation of the coupled influence mechanism of impurities on CO_2_ phase behavior and corrosion and the establishment of corrosion prediction models based on actual operational conditions have become key scientific issues for ensuring the large-scale application of CCUS technology [3,25].

Therefore, systematically reviewing the alterations caused by impurity gases (such as H_2_O, O_2_, SO_2_, H_2_S, NO_2_, and non-condensable gases) to the thermodynamic/transport properties of sCO_2_ pipeline fluid, deeply analyzing the complex electrochemical corrosion mechanisms occurring at the interface with pipeline steel (particularly the synergistic effects between impurities), and clarifying critical impurity concentration thresholds and engineering control standards are crucial for accurately assessing pipeline service risks, optimizing transportation process parameters, and formulating economically effective corrosion prevention and control strategies. This review focuses on the core challenges posed by impurities to the CO_2_ pipeline transportation system. It aims to comprehensively integrate the latest domestic and international research findings and clarify the influence patterns of different impurities (single and coexisting) on phase stability, corrosion behavior characteristics (uniform corrosion, pitting, and corrosion stress cracking (CSC)), and pipeline integrity, thereby providing a solid theoretical foundation and engineering guidance for the safe and efficient use of CO_2_ pipeline transportation technology in the large-scale commercial application of CCUS.

## 2. Impact of Impurities on Pipeline Fluid Properties

### 2.1. Key Changes in Phase Behavior and Density

Impurity gases significantly impact pipeline transport efficiency by modulating the thermodynamic and transport properties of CO_2_. Regarding phase behavior and density, non-condensable gases (e.g., N_2_, CH_4_, and H_2_) elevate the critical pressure (increasing from 7.38 MPa to 7.55 MPa when the N_2_ concentration reaches 7%), thereby increasing the operational difficulty of maintaining the supercritical state. Furthermore, light gases like H_2_ significantly reduce the density (a decrease of >50% at 10% H_2_ concentration) and expand the phase envelope (critical temperature < −56.7 °C), diminishing the transport capacity per unit volume [26,27]. In contrast, acidic gases (SO_2_, H_2_S) raise the critical temperature, which favors the stability of the dense-phase fluid at lower temperatures [28]. The molecular weight of the impurity gas has a notable impact on density: heavier gases (e.g., Ar) increase density, while lighter gases decrease it. This is directly linked to the economic feasibility of pipeline transportation [29,30,31].

### 2.2. Fluidity and Transportation Energy Consumption

Impurities significantly influence the fluidity and energy consumption of CO_2_ pipeline transportation. During the compression stage, impurity gases markedly increase energy consumption: for every 1% increase in the concentration of O_2_, N_2_, and H_2_, the compression work increases by approximately 2.5%, 3.5%, and 4.5%, respectively, with the lower molecular weight H_2_ having a particularly pronounced effect. This is primarily because non-condensable gases occupy pipeline volume, increasing compression demands [28,32]. Furthermore, impurities affect pressure drop by altering viscosity and flow velocity. For instance, H_2_O, SO_2_, and NH3 cause an increase in viscosity, which further elevates flow resistance and pressure drop, thereby reducing transport efficiency. Conversely, light gases decrease fluid viscosity but simultaneously increase flow velocity, leading to a significant rise in frictional resistance and a consequent increase in energy consumption. Therefore, optimizing impurity control is essential in pipeline system design to maintain operational economy [28,33].

## 3. Threats of Impurities to Pipeline Integrity

### 3.1. Core Role of H_2_O

**Figure 2:**
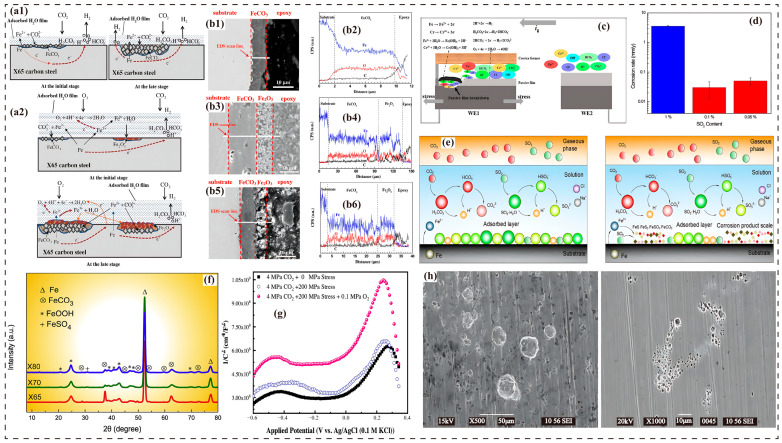
(**a1**,**a2**,**b1**–**b6**) Schematic diagrams of corrosion mechanisms and SEM images of corrosion products for X65 carbon steel in H_2_O-saturated sCO_2_ phase without and with O_2_ [34]; (**c**) corrosion mechanism diagram of 13Cr in the presence of O_2_ [36]; (**d**) effect of SO_2_ concentration on the corrosion rate of carbon steel in sCO_2_ phase at a CO_2_ partial pressure of 8 MPa, a temperature of 50 °C, water content of 650 ppm, and an exposure time of 24 h [35]; (**e**) corrosion mechanism of X80 steel in a CO_2_–H_2_O environment containing SO_2_ [59]; (**f**) XRD spectra of X65 steel exposed to 10 MPa water-saturated sCO_2_ containing 3.0% O_2_ + 100 ppm SO_2_ [60]; (**g**) Mott–Schottky curves of 13Cr stainless steel in solution containing 4 MPa CO_2_ (with/without 0.1 MPa O_2_) under different stress conditions [36]; (**h**) scanning electron microscopy images of corrosion surfaces of samples exposed to liquid CO_2_ for 24 h at a CO_2_ partial pressure of 8 MPa and a temperature of 50 °C in a 0.1% SO_2_ environment and a 0.05% SO_2_ environment [35].

In sCO_2_ pipeline systems, H_2_O is the core factor initiating corrosion. By reacting with CO_2_ to form carbonic acid, it lowers the environmental pH to 3–4, initiating the electrochemical corrosion process. This forms a conductive water film on the steel surface, accelerating anodic dissolution and cathodic reduction. When the H_2_O concentration is below the critical value, only slow uniform corrosion occurs, with rates potentially below 0.025 mm/y. Once solubility is exceeded, particularly in the presence of impurities like SO_2_ or O_2_, a free water phase separates, and corrosion intensifies sharply, with rates potentially rising to 7 mm/y. This modulates the morphology of corrosion products and the susceptibility to localized corrosion, initiating pitting, CSC, and the formation of non-protective corrosion layers (e.g., FeCO_3_), significantly increasing the risk of material degradation (Figure 2a) [34]. Aqueous phase separation becomes a major driver for wellbore corrosion, making strict water control essential to ensure system integrity [35,36].

The phase distribution inside the pipeline is strongly correlated with water content: at low water content, the CO_2_ phase dominates, inhibiting the formation of a continuous water film; after exceeding the supercritical threshold, the aqueous phase accumulates at the bottom, forming corrosion hotspots [37,38,39,40]. For J55 steel, the surface becomes fully water-covered at water content > 75%, leading to corrosion product film rupture and initiating pitting. Under conditions with impurities, the localized corrosion rate of X80 steel in the water-rich phase can be up to 10 times that in the CO_2_-rich phase [41,42]. The critical water content is regulated by temperature and pressure parameters and remains controversial: reported thresholds vary significantly (100–1000 ppm), stemming from differences in experimental conditions (e.g., temperature gradients of 25–60 °C) [43,44,45,46]. For X65 steel at 50 °C/8 MPa, the uniform corrosion rate fluctuates between 0.01 and 0.4 mm/y, and the intensity of localized corrosion can be up to 14 times that of uniform corrosion [43,47,48]. The presence of H_2_O also exacerbates corrosion through synergistic effects (e.g., interacting with SO_2_/O_2_ impurities to promote acid regeneration cycles) and modulates corrosion product morphology: at high concentrations, the product layer becomes porous and cracked, leading to continuous exposure of the substrate [17,49]. There is an urgent need to establish standardized testing methods and analyze the multi-field coupling mechanisms to improve pipeline integrity management.

### 3.2. Impact of O_2_

During CO_2_ pipeline transportation, O_2_, as a common impurity, exhibits a complex “concentration dependence” in its effect on the corrosion behavior of pipeline steels [50]. O_2_ participates in the cathodic reduction reaction (O_2_ + 2H_2_O + 4e^−^ → 4OH^−^), inhibiting the formation of a dense, protective FeCO_3_ film, thereby accelerating the uniform corrosion rate of steel [51,52]. This influence demonstrates distinct concentration-dependent characteristics, which specifically manifest as follows.

(1) Corrosion Promotion by Low-Concentration O_2_

In water-saturated sCO_2_ environments, trace O_2_ (1.5 ppm) can increase the corrosion rate of carbon steel and 13Cr stainless steel beyond 100 mm/y [43]. When the O_2_ concentration reaches 200 ppm, the corrosion rate of X70 steel significantly rises from a baseline of 0.0577 mm/y to 0.09 mm/y [53]. This acceleration stems from O_2_ disrupting the integrity of the protective FeCO_3_ film and promoting the formation of Fe^3+^ oxides [14,53]. Tang et al. [34] found, through comparative experiments, that an FeCO_3_–Fe_2_O_3_ bilayer structure forms on the surface of X65 steel in O_2_-containing environments, wherein the porous Fe_2_O_3_ film acts as a channel for corrosion propagation (Figure 2b). This finding aligns with the mechanism proposed by Dugstad et al. [54], where the O_2_–Fe^2+^ reaction leads to local dissolution of the FeCO_3_ film.

(2) Corrosion Inhibition and Passivation Effect at High O_2_ Concentrations

However, when the O_2_ concentration increases to 5700 ppm, the corrosion rate becomes lower than that in an oxygen-free environment [43]. This concentration dependence may be related to the regulation of the corrosion product film structure by O_2_ partial pressure. At low partial pressures (<500 ppm), O_2_ hinders the stable deposition of FeCO_3_ nuclei, resulting in the formation of a loose and porous Fe_2_O_3_ film (Figure 2b) [34,46], whereas, at high partial pressures (e.g., 1000 ppm), O_2_ promotes the formation of dense oxides such as Fe_3_O_4_, FeOOH, and Fe_2_O_3_, reducing the corrosion rate of X70 steel to 0.03 mm/y. X65 steel exhibits a passivation tendency, and, for 13Cr steel, it helps improve the denseness of the Cr_2_O_3_/Cr(OH)_3_ film (Figure 2g) [36,46,49]. Although uniform corrosion is suppressed, O_2_ concentrations > 500 ppm can induce localized pitting corrosion (the pitting rate of X65 steel at 1000 ppm O_2_ reaches 3 mm/y) due to galvanic corrosion caused by inhomogeneous oxide films [14].

From the perspective of corrosion kinetics, the intervention of O_2_ in electrochemical processes exhibits stage-specific characteristics [55]. Studies on dynamic sCO_2_ water-rich phases indicate that O_2_ significantly accelerates the corrosion process in the initial reaction stage by promoting the oxidation of Fe^2+^ to Fe^3+^. However, with the accumulation of Fe(OH)_3_ and Fe_2_O_3_, their diffusion barrier effect leads to the enrichment of Fe^2+^ on the metal surface, which conversely aids the reconstruction of the protective FeCO_3_ layer. This phenomenon is particularly evident in N80 steel, whose corrosion rate shows a trend of initially increasing and subsequently decreasing with reaction time [56]. However, studies on X65 steel reveal that O_2_ causes the corrosion product film to transition from dense FeCO_3_ to a multiphase mixed structure (FeCO_3_–Fe_2_O_3_). In this structure, the FeCO_3_ regions impede O_2_ permeation, while the Fe_2_O_3_ regions become channels for ion migration, ultimately leading to a significant increase in the localized pitting rate [34,57]. This phase separation effect effectively explains why the average corrosion rate decreases in some studies while the pitting risk increases dramatically [19,46,58]. Under the synergistic action of stress and crevices, O_2_, while promoting the repair of the passive film outside crevices on 13Cr, enlarges the potential difference and exacerbates anodic dissolution inside the crevices (Figure 2c) [36].

The synergistic effect of temperature and O_2_ further complicates the corrosion behavior. The formation and growth of FeCO_3_ primarily consist of two stages: nucleation and particle growth. The nucleation process results in a dense and complete FeCO_3_ product film. An elevated temperature promotes the rate of the nucleation process and simultaneously reduces the solubility product of FeCO_3_, facilitating FeCO_3_ deposition and enhancing the protective effect of the corrosion product film on the substrate. However, an increased temperature also leads to a higher pH value and intensifies the anodic and cathodic electrochemical reactions on the steel surface, promoting the occurrence of corrosion reactions [61]. Influenced by these contradictory factors, the corrosion rate typically peaks between 60 and 90 °C [62,63].

For supercritical CO_2_ environments containing O_2_, a comprehensive, multi-level protection system must be established:

① Material upgrade serves as the fundamental measure. Material selection should be graded according to O_2_ concentration: low-Cr steels with excellent cost-effectiveness can be chosen for low-concentration conditions (O_2_ < 500 ppm); 13Cr stainless steel is required for medium–high concentrations (O_2_ ~ 1000 ppm); and 2205 duplex stainless steel is necessary for extremely harsh environments [14]. A cost-effective alternative can involve carbon steel combined with an epoxy resin liner [64];

② Use of corrosion inhibitor technology is an economically effective chemical protection method. Oxidizing inhibitors (e.g., molybdates) can preferentially react with O_2_ to form a passive film; adsorption-type inhibitors (e.g., imidazoline derivatives) function by forming a monomolecular layer that blocks corrosive media [49,65]. Their injection method (continuous or pulse) and concentration need to be dynamically optimized based on system operating conditions and must be coordinated with dehydration processes to prevent moisture from reducing their efficiency [36,66];

③ Process optimization and intelligent monitoring constitute an active defense barrier. Strictly maintaining the supercritical state and maintaining reasonable flow velocities can significantly inhibit corrosion. Intelligent monitoring systems integrating laser oxygen analyzers and electrochemical noise sensors enable real-time, precise measurement of O_2_ concentration and early warning of corrosion risks, providing crucial data support for proactive intervention [29].

### 3.3. Impact of SO_2_

In CO_2_ transportation pipelines, the corrosion behavior of SO_2_ is jointly regulated by its dissolution characteristics and electrochemical reactions [61,67,68]. Residual moisture dissolves SO_2_ to participate in cathodic ionization, generating sulfides under oxygen-free environments (Figure 2e) [59]. XRD spectra indicate similar types of corrosion products for X65, X70, and X80 steels, suggesting that steel grade has no significant influence on the phase composition of corrosion products (Figure 2f) [60].

The corrosion exhibits notable concentration dependence. An SO_2_ concentration of 1% significantly increases the corrosion rate of carbon steel (approximately 3.5 mm/y). SO_2_ concentrations below 0.1% cause minor corrosion that stabilizes over time, but 0.05% SO_2_ in liquid CO_2_ can initiate localized corrosion (reaching rates of 2.4 mm/y). High SO_2_ concentrations lead to the formation of H_2_SO_3_ and H_2_SO4, causing localized pH drops and corrosive product formation, while low concentrations produce insufficient reaction products to continuously damage the steel surface (Figure 2d,h) [35].

Under oxygen-free conditions, low concentrations of SO_2_ (30–300 ppm) can form a protective FeS film that inhibits corrosion [15,69]. However, when the concentration rises to 0.08%, the corrosion rate of X70 steel in sCO_2_ reaches 3.48 mm/y, and it increases to 3.70 mm/y when coexisting with 0.33% O_2_. This exacerbation stems from SO_2_ hydrolysis creating an acidic environment that damages protective layers [64,70]. Even trace amounts of SO_2_ exacerbate pitting corrosion in pipeline steels due to the initial formation of weakly adherent FeS/FeS_2_ films, whose protective efficacy decreases with increasing concentration [19,70,71,72]. Exposure to 500 ppm SO_2_ elevates the corrosion rate of X70 steel in water-saturated sCO_2_ to 1.10 mm/y [53].

Environmental conditions significantly modulate the corrosion process. SO_2_ reduces water solubility in sCO_2_, promoting the precipitation of a weakly acidic H_2_SO_3_ water film. Increasing the SO_2_ concentration causes a sharp decline in the pH of the separated aqueous phase (a 31.1% drop at 2000 ppm), resulting in a pitting rate of 0.08 mm/y for X65 steel [61,73]. While the synergistic effect of O_2_ and SO_2_ yields a relatively minor increase in corrosion rate, significant localized corrosion is still observed in high-pressure liquid CO_2_ (containing 650 ppm H_2_O + 0.05% SO_2_) [35].

For corrosion environments containing SO_2_, comprehensive protection strategies should be adopted:

① Material upgrade forms the basis for resisting SO_2_ corrosion. Material selection must be based on SO_2_ concentration and operating conditions: 316L stainless steel is suitable for environments with SO_2_ < 100 ppm; however, when the SO_2_ concentration is high or it coexists with O_2_, 2205 or 2507 duplex stainless steels with superior pitting resistance should be selected [55]. An economical alternative can employ low-Cr steel (e.g., 3Cr) combined with a PTFE liner to effectively isolate the corrosive medium [64];

② Use of corrosion inhibitor technology is a key chemical method for corrosion control. Reducing inhibitors like thiosulfate can react with SO_2_ to generate protective films; amine-based inhibitors function by neutralizing the formed sulfurous acid [68]. Employing a composite inhibitor system (e.g., a combination of imidazoline and molybdate) synergistically with pH regulators (maintaining the system pH at 5.5–6.5) can achieve more stable and efficient protection. The dosing strategy needs dynamic adjustment based on fluctuations in SO_2_ concentration [49];

③ Process optimization and intelligent monitoring constitute a systematic line of defense. The core is ensuring that the fluid remains in a stable supercritical state and implementing strict dehydration (H_2_O < 50 ppm) to fundamentally inhibit the formation of H_2_SO_3_. In terms of fluid design, adopting low flow velocities and structures that improve the flow field’s uniformity can prevent local enrichment of SO_2_ [60]. Simultaneously, utilizing high-precision equipment such as ultraviolet fluorescence SO_2_ analyzers for real-time monitoring and establishing a tiered early warning mechanism are essential guarantees for the early detection and proactive management of corrosion risks [64,74].

### 3.4. Impact of H_2_S

**Figure 3:**
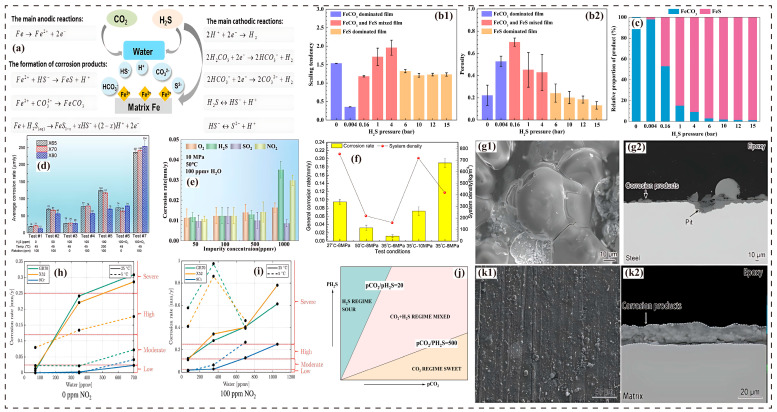
(**a**) Main corrosion reactions of steel in the sCO_2_–H_2_O–H_2_S system [12]; (**b1**,**b2**,**c**) structural tendency of corrosion product films and calculated relative proportions of surface-generated FeCO_3_ and FeS products on N80 steel in different sCO_2_/H_2_S environments (8 MPa CO_2_, 80 °C, 72 h) [80]; (**d**) average corrosion rates of X65, X70, and X80 steel in H_2_O-saturated sCO_2_ flow with the presence of H_2_S and/or O_2_ under different temperatures and rotation speeds for 120 h [77]; (**e**) variation in corrosion rate of X52 steel in a high-pressure CO_2_ environment influenced by impurity type and concentration under different factors [96]; (**f**) relationship between corrosion rate and system density of X65 carbon steel under different temperature–pressure conditions (H_2_S 1000 ppm, H_2_O 10 g) [12]; (**g1**,**g2**) SEM surface morphology and cross-sectional backscattered electron images of corrosion film on X65 steel in an sCO_2_–NO_2_ system at 8 MPa and 50 °C [74]; (**h**,**i**) relationship between NO_2_ corrosion rate and water concentration (NO_2_ content, 0 ppm and 100 ppm; temperature, 5 °C and 25 °C; red dashed horizontal line represents the corrosion severity threshold for carbon steel defined by the National Association of Corrosion Engineers (NACE) standard) [90]; (**j**) H_2_S corrosion control zoning diagram [97]; (**k1**,**k2**) SEM surface morphology of corrosion film on X65 steel in a water-saturated CO_2_ (wsCO_2_)–H_2_S system at 8 MPa and 50 °C [68].

**Figure 4:**
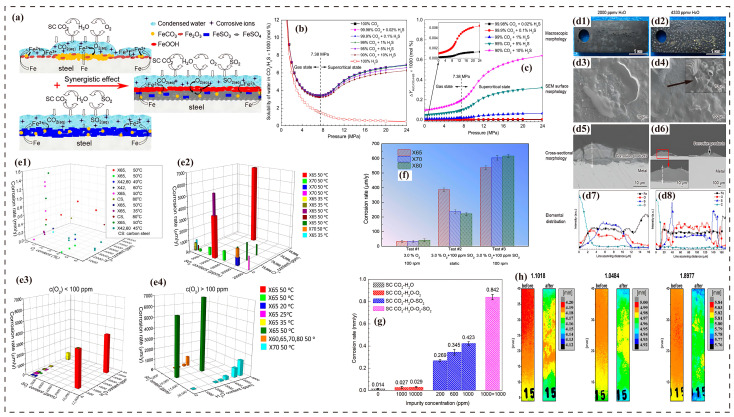
(**a**) Schematic models of corrosion and film characteristics in water-saturated sCO_2_–H_2_O–O_2_, sCO_2_–H_2_O–SO_2_, and sCO_2_–H_2_O–O_2_–SO_2_ systems [19]; (**b**,**c**) variation in H_2_O solubility in CO_2_ and/or H_2_S with pressure and the corresponding precipitation amount [41]; (**d1**–**d6**) condition of X65 steel after 72 h of exposure in an sCO_2_–H_2_O–O_2_–H_2_S–SO_2_ environment at 10 MPa and 50 °C (**d1**,**d3**,**d5**,**d7**) under 2000 ppmv water vapor conditions and (**d2**,**d4**,**d6**,**d8**) under 4333 ppmv water vapor conditions [41]; (**e1**) corrosion rate of carbon steel in an sCO_2_ environment within a pressure range of 8–15 MPa and a temperature range of 35–80 °C under different water and oxygen contents [25]; (**e2**–**e4**) influence of SO_2_, O_2_, and H_2_O on the corrosion rate of different steels in an sCO_2_ environment within a pressure range of 8–10 MPa at different temperatures [60]; (**f**) average corrosion rate of X65 steel after 120 h of exposure in water-saturated CO_2_ flow at 45 °C and 10 MPa under different impurity contents and rotation speeds [60]; (**g**) corrosion rate of X70 steel after 120 h of exposure in a water-saturated sCO_2_ system containing O_2_ and/or SO_2_ at 10 MPa and 50 °C [19]; (**h**) thickness distribution comparison of pure iron 1.1018, pipeline steel L290NB 1.0484, and pipeline steel L485MB 1.8977 before and after exposure to a CO_2_ environment with impurities (660 ppm SO_2_, 0.66% O_2_, 2.05% H_2_O, 5 °C, 120 h) [71].

In environments where CO_2_ and H_2_S coexist, metal corrosion is synergistically regulated by the gas concentration ratio, partial pressure ratio, and environmental parameters [64,75,76]. H_2_S concentration exhibits a typical nonlinear effect: low concentrations (<500 ppm) significantly accelerate uniform corrosion but inhibit localized corrosion [77]. In sCO_2_, 50 ppm H_2_S increases the corrosion rate of X65 steel from 0.17 mm/y to 0.24 mm/y, while raising the H_2_S concentration to 100 ppm has a limited further effect on the corrosion rate, suggesting that 50 ppm may be the threshold concentration for the formation of a protective FeS layer (Figure 3d) [77]. The increase is more pronounced in the presence of an aqueous phase, where the corrosion rate rises from 8.46 mm/y to 15.48 mm/y at the same concentration. This is because H_2_S alters the phase equilibrium of the CO_2_–H_2_O system, promoting the separation of an aqueous phase that provides an electrolyte environment for corrosion (Figure 3a and Figure 4b,c) [12,41]. Furthermore, H_2_S modifies the water adsorption characteristics of the steel surface, promoting water film coverage and intensifying electrochemical corrosion. The mechanism for localized corrosion inhibition is related to the H_2_S-promoted formation of a dense FeCO_3_–FeS mixed film. This mixed film offers low protectiveness and is characterized by being porous and micro-cracked (Figure 3k) [57,68,78,79].

At medium-to-high concentrations (>500 ppm), H_2_S exhibits a bimodal effect. When the H_2_S partial pressure rises to 0.0004 MPa (approximately 40 ppm), the corrosion rate of N80 steel in 8 MPa sCO_2_ increases from 4.23 mm/y to 4.61 mm/y. However, when the partial pressure reaches 0.4 MPa (approximately 40,000 ppm), the rate decreases to 0.72 mm/y (Figure 3b,c) [80]. This transition is governed by the CO_2_/H_2_S partial pressure ratio. A ratio <200 promotes H_2_S-dominated formation of an FeS film. A ratio >500 favors CO_2_-dominated formation of an FeCO_3_ film (Figure 3j) [76,81,82]. When the CO_2_ pressure is fixed, increasing the H_2_S concentration causes the corrosion rate to first rise and then fall. Conversely, at a fixed H_2_S pressure, increasing the CO_2_ pressure ratio continuously elevates the corrosion rate and drives the transition of corrosion products from FeS to FeCO_3_ [81,83,84]. The mixed film structure is loose and prone to spalling, exacerbating uniform corrosion.

When the H_2_S concentration exceeds 1000 ppm, the anomalous increase in corrosion rate is primarily driven by the abrupt changes in CO_2_ physical properties near the critical point, while density predominantly influences corrosion trends only in non-critical regions. The corrosion products shift from being dominated by FeCO_3_ to FeS or FeCO_3_–FeS mixed structures (Figure 3f) [12]. Research findings exhibit discrepancies. For instance, Sun et al. [80] observed that H_2_S partial pressure regulates corrosion products in a stepwise manner (FeCO_3_ at <0.016 MPa, mixed at 0.016–0.4 MPa, and predominantly FeS at >0.4 MPa), which aligns with Choi [85]. In contrast, Wei et al. [32] found that X65 steel forms an FeS–FeCO_3_ bilayer film, which exacerbates localized corrosion due to galvanic effects. These differences may originate from variations in alloy composition and phase conditions. Addressing H_2_S corrosion requires establishing a comprehensive system that considers both general corrosion and localized cracking:

① Material upgrade serves as the foundation for ensuring safety. For low H_2_S concentrations (<50 ppm), sulfur-resistant carbon steel with controlled hardness can be selected; for medium–high concentrations or harsh service conditions, corrosion-resistant alloys such as duplex stainless steels or nickel-based alloys must be employed [32,86];

② Corrosion inhibitor technology is key to corrosion control. Film-forming inhibitors (e.g., quaternary ammonium salts, imidazoline derivatives) can form an adsorptive barrier layer on the metal surface. To ensure their effectiveness, inhibitor selection must be validated through autoclave testing and used in combination with oxygen scavengers to maintain the dissolved oxygen concentration at an extremely low level, thereby preventing degradation of the inhibitor film [83,84];

③ Process optimization aims to eliminate the root causes of corrosion. The core lies in stringent process control, including reducing the water concentration to <30 ppm through deep dehydration and precisely regulating the temperature, flow velocity (recommended 1–2 m/s), and system pH (6–7.5) in order to prevent aqueous phase condensation, mitigate mechanical erosion of inhibitor films, and inhibit the formation of localized acidic environments [41,64];

④ Monitoring and early warning systems constitute a safeguard for achieving proactive defense. It is necessary to integrate real-time monitoring technologies such as online H_2_S analysis and electrochemical noise analyzers and establish a tiered alarm mechanism [17]. Combining periodic non-destructive testing with data-driven digital twin models enables accurate prediction of the Sulfide Stress Cracking (SSC) risk, facilitating early warning and proactive intervention [3].

### 3.5. Impact of NO_2_

In CO_2_ pipeline transportation, NO_2_ is a highly corrosive impurity that accelerates aqueous phase separation and generates HNO_3_ (3NO_2_ + H_2_O → 2HNO_3_ + NO) when coexisting with H_2_O, significantly reducing the local pH, damaging the protective FeCO_3_ film, and inducing pitting corrosion (Figure 3g) [17,70,74,87,88]. Studies confirm that NO_2_-containing environments lead to thinning of corrosion product films and exacerbate uniform corrosion [70,89]. The corrosion exhibits strong concentration dependence. The corrosion rate of X52 steel at 5 °C increases fivefold compared with 25 °C (0.016 → 0.299 mm/y). At 50 °C, as the NO_2_ concentration rises from 50 ppmv to 1000 ppmv, the corrosion rate shows an initial slow increase followed by a sharp rise (Figure 3e). In sCO_2_, 100 ppm NO_2_ can elevate the corrosion rate of carbon steel to 11.6 mm/y (twice that of SO_2_ at the same concentration) [67,87,90].

The corrosion mechanism of NO_2_ exhibits multiple characteristics. HNO_3_ erosion reduces the film’s denseness and creates voids, and synergy with O_2_/SO_2_ further amplifies the risk [17]. Low temperatures (e.g., 5 °C) significantly intensify the corrosion effect due to the reduced water solubility leading to an increase in the acid concentration. The corrosion rate of X65 steel at 5 °C is 3–4 times higher than that at 25 °C (Figure 3h,i) [64,74,90]. Under prolonged exposure, NO_2_ not only accelerates uniform corrosion but also initiates localized pitting and SCC, particularly in dynamic flow fields where the uneven deposition of corrosion products creates stress concentration points [55,74,88]. Microscopic analysis revealed that NO_2_ alters FeCO_3_ morphology, reduces film denseness, and enhances galvanic effects, leading to synergistic development of pitting and uniform corrosion with far greater severity than other gases [17,70]. Therefore, strict control of NO_2_ concentration and water content is essential to suppress corrosion progression.

### 3.6. Impact of Non-Condensable Gases

Non-condensable gases (N_2_, O_2_, Ar, CH_4_, and H_2_) promote the separation of free aqueous phase by reducing water solubility in sCO_2_, inducing electrochemical corrosion [91]. The typical volumetric fraction threshold is 4%; beyond this limit, water separation occurs even at water content < 500 ppm [25,90]. CH_4_ significantly alters the phase behavior: 10% concentration increases the critical pressure to 14.75 ± 0.25 MPa, requiring higher operating pressures; 20% concentration reduces the water solubility, triggering localized corrosion. When coexisting with H_2_S, it may promote the formation of non-protective FeS films, with synergistic effects from N_2_ further exacerbating the risk [57,92,93].

Ar lowers the critical temperature and raises the critical pressure (reaching 7.55 MPa at 4.5%), increasing the operational pressure. A concentration of 20% modifies fluid properties and reduces the protectiveness of corrosion product films [94]. N_2_ is one of the most corrosion-promoting impurities. A concentration of 7% lowers the CO_2_ critical temperature to 30.37 °C and raises the critical pressure to 7.55 MPa, while 10% concentration reduces the water solubility by 30%, forming corrosive liquid films, accelerating O_2_ diffusion to enhance cathodic depolarization rates, and doubling the corrosion rate of carbon steel [49,57,92,95]. CO may generate formic acid, lowering the pH, and at 0.2% concentration catalyzes hydrogen permeation, increasing the hydrogen embrittlement risk [93].

Additionally, maintaining a supercritical state requires elevated pressures, increasing compression costs and accelerating SCC [25]. At H_2_ concentrations >0.5%, hydrogen atoms permeate the steel matrix, inducing SCC. In environments with 4% H_2_, the hydrogen permeation flux of X65 steel triples, with the SCC susceptibility index reaching 0.28 (critical value 0.25) under dynamic pressure. High pressures (>8 MPa) and low temperatures (<10 °C) exacerbate this risk [93,94].

### 3.7. Corrosion Synergistic Effects

During sCO_2_ pipeline transportation, the synergistic effects between impurity gases and H_2_O exert complex and nonlinear influences on steel corrosion behavior, with mechanisms extending far beyond the sum of individual impurity effects (Table 1) [39]. As the core initiator of corrosion, H_2_O not only enables electrochemical reactions but also regulates the phase distribution. At low water content (<1500 ppm), corrosion is mild, but exceeding the critical threshold (e.g., 1500–2000 ppm) leads to free aqueous phase separation, causing corrosion rates to surge from <0.025 mm/y to 7 mm/y and triggering localized pitting and SCC [10,25,98]. This aqueous phase separation is significantly amplified under impurity coexistence; for instance, SO_2_ can reduce the critical water content threshold from 1000 ppm to 50 ppm, accelerating the formation of acidic electrolyte films [41,54].

Interactions between impurities exhibit significant concentration dependence. Trace amounts of O_2_ can elevate the corrosion rate of carbon steel beyond 100 mm/y by disrupting the protective FeCO_3_ film and promoting the formation of Fe^3+^ oxides [14,43]. When O_2_ coexists with SO_2_ (e.g., 200 ppm O_2_ + 500 ppm SO_2_), their synergistic effect drastically increases the corrosion rate to 20.47 mm/y—far exceeding the sum of individual impurity effects (18.64 mm/y)—as O_2_ catalyzes the conversion of SO_2_ into highly corrosive H_2_SO4, which reacts with steel to form FeSO4 and FeOOH (Figure 4a,g) [19,55,99]. This effect is further intensified at elevated temperatures (60–90 °C) due to enhanced oxidation of Fe^2+^, leading to porous Fe_2_O_3_ films that compromise the continuity of the product layer [34,63]. In sCO_2_–SO_2_–O_2_–H_2_O systems, environmental factors (e.g., impurity concentration, flow velocity) exert a far greater influence on corrosion than minor variations in material composition (Figure 4f,h) [60,71]. Notably, while high O_2_ concentrations (>500 ppm) inhibit uniform corrosion, they induce localized pitting (with pitting rates reaching 3 mm/y for X65 steel) due to galvanic corrosion caused by inhomogeneous oxide films [14,49].

The corrosive effect of SO_2_ is achieved through hydrolysis, forming H_2_SO_3_/H_2_SO4. When its concentration exceeds 100 ppm, it significantly reduces the aqueous phase pH (e.g., 2000 ppm SO_2_ causes a 31.1% pH drop), leading to a pitting rate of 0.08 mm/y for X65 steel (Figure 4e) [47,61,73]. When coexisting with NO_2_, the oxidation of SO_2_ to H_2_SO4 accelerates, increasing the corrosion rate by 40% [70,74]. Low concentrations of H_2_S (<500 ppm) accelerate corrosion (50 ppm H_2_S increases the corrosion rate of X65 steel from 0.17 mm/y to 0.24 mm/y), while high concentrations (>1000 ppm) may form FeS films that inhibit uniform corrosion but pose a risk of localized pitting [57,78,80]. When H_2_S coexists with O_2_, elemental sulfur formation exacerbates localized corrosion, driving the corrosion rate of X70 steel beyond 7 mm/y [58,100]. The redox reaction between SO_2_ and H_2_S (2H_2_S + SO_2_ → 3S + 2H_2_O) also generates sulfur deposits. When SO_2_ > 200 ppm and H_2_S > 500 ppm, the sulfur generation rate reaches 1.2 kg/d·km, causing equipment blockage and a pressure drop (X70 steel exhibits 38 g/m^2^ deposit accumulation in O_2_-containing environments over 480 h, with a 15% pressure increase) (Figure 4d,g) [49,58,70,100]. The reaction between deposited sulfur and Fe forms FeS_2_, with a 40% volumetric expansion rate, accelerating crack propagation [12]. In environments where sCO_2_–H_2_O–O_2_–H_2_S–SO_2_ coexist, the formation of H_2_SO4 and S makes the system over 10 times more corrosive than with single impurities. H_2_O at 4333 ppm fully covers the metal surface, accelerating ion diffusion and electrochemical reactions. The product layer thickness surges to 150 μm, with localized laminated FeSO_3_ crystals and wormhole-like sulfur-rich phases (Figure 4d) [41].

NO_2_ is the most hazardous impurity, with even 100 ppm capable of driving the corrosion rate of carbon steel to 11.6 mm/y. Its mechanism involves the reaction 3NO_2_ + H_2_O → 2HNO_3_ + NO, generating a strong acid that disrupts the integrity of corrosion product films [70,87]. When synergized with O_2_ (e.g., 100 ppm NO_2_ + 1000 ppm O_2_), the localized corrosion rate surges to 6.8 mm/y, with low-temperature (5 °C) environments amplifying this effect by 3–4 times [74,90]. Non-condensable gases (e.g., N_2_, CH_4_) indirectly exacerbate corrosion by reducing water solubility (7% N_2_ decreases solubility by 30%), promoting aqueous phase separation. Two-phase flow corrosion occurs when their total volume fraction exceeds 4% [25,93,95].

Temperature–pressure parameters exhibit strong coupling effects with impurities. At high temperatures (80 °C) and H_2_S partial pressures exceeding 0.4 MPa, corrosion products shift from predominantly FeCO_3_ to FeS, reducing the corrosion rate from 4.61 mm/y to 0.72 mm/y [80]. Conversely, when the pressure rises to 10 MPa, non-condensable gases necessitate higher operating pressures, accelerating SCC [94]. This multi-field coupling mechanism indicates that corrosion control requires integrated management of impurity concentration thresholds (e.g., O_2_ < 1000 ppm, SO_2_ < 100 ppm, NO_2_ < 1.5 ppm), water content (<650 ppm), and material selection to disrupt the autocatalytic corrosion cycles triggered by synergistic effects [19,70,86].

## 4. Engineering Standards for Impurity Control

### 4.1. Industry Specifications and Thresholds

Pipeline transportation has become the preferred solution for large-scale onshore CCS/CCUS projects due to its continuity, stability, economic efficiency, and technological maturity [50,101,102]. The total length of the existing global CO_2_ pipeline network exceeds approximately 10,000 km, of which over 8000 km are located in the United States and are primarily used for CCUS with Enhanced Oil Recovery (CCUS-EOR), while Canada operates more than 300 km. To achieve carbon neutrality, China requires the construction of over 17,000 km of CO_2_ pipelines, though only several hundred kilometers have been built so far. The core technology involves pressurizing gaseous CO_2_ to a supercritical state (typically >8 MPa) to avoid two-phase flow and optimize transportation costs. In contrast, offshore pipelines have not yet achieved large-scale development due to construction challenges and high costs, with only limited-distance subsea pipelines deployed in a few projects [101,103,104,105].

The existing pipeline network in North America exhibits significant regional variations, primarily categorized into three types. Type II pipelines, which are the most widely deployed, adhere to strict gas quality specifications (often utilizing natural carbon sources, operating at 1.72–15.17 MPa). Type I pipelines are short-distance, dedicated pipelines for point-to-point projects. Type III pipelines are used in regional blended networks with relatively lenient gas quality standards, requiring additional corrosion mitigation measures. Due to substantial differences in gas composition, Type III networks face challenges in interoperability with Type II systems [50,64,106]. North America lacks unified industry standards, with pipeline gas quality primarily governed by commercial agreements. Existing projects maintain >95% CO_2_ content to align with EOR requirements. In Europe, large-scale subsea pipeline networks (planned mileage: 30,000–150,000 km) are under development [94,107]. Ever since the DYNAMIS project proposed initial gas quality requirements in 2007, subsequent initiatives, such as Ecofys, Pace CCS, Porthos, and Norway’s Northern Lights, have established differentiated specifications, significantly advancing the standardization of CO_2_ pipeline transportation (Table 2) [108].

### 4.2. Synergistic Impurity Control Strategies

The management of impurities in sCO_2_ pipelines requires a hierarchical control system. Dehydration serves as the primary step, where reducing the H_2_O concentration to <50 ppm via molecular sieve adsorption effectively disrupts corrosive chain reactions [70]. Experimental data indicate that, when the water content exceeds 1500 ppm, the corrosion rate of X65 steel surges from 0.025 mm/y to 7 mm/y, while dehydration to 50 ppm reduces the corrosion rate by 98% [41]. Oxidizing impurities necessitate dual limits (maintaining O_2_ < 100 ppm and NO_X_ < 1.5 ppm suppresses strong acid formation, thereby mitigating effective corrosion rates) [19,70]. Special attention must be paid to scenarios where the O_2_ concentration exceeds 500 ppm, as NO_X_ levels as low as 50 ppm can trigger pitting corrosion through acidification reactions [53,74].

Economic optimization requires balancing compression energy consumption and specific impurity risks. The total volume of non-condensable gases should be <4 vol%; when their proportion increases from 2% to 6%, compression work rises by 35% and transportation costs increase by 28% [93]. Specific control targets include H_2_ < 0.1% (to prevent hydrogen embrittlement), N_2_ < 3% (to maintain phase stability), and CH_4_ < 0.9% (to avoid two-phase flow) [94].

To mitigate sulfur deposition risks, synergistic control of H_2_S (<200 ppm) and SO_2_ (<100 ppm) is necessary to prevent the formation of elemental sulfur that could clog pipelines [49,70]. For existing sulfur blockages, treatment with 2–5 vol% carbon disulfide solvent can be applied, but the contact time must be limited to <4 h [70].

Dynamic regulation should optimize thresholds based on operational parameters. Under high pressures (>10 MPa), the O_2_ threshold should be reduced to 50 ppm, while at elevated temperatures (>60 °C), the H_2_S limit can be relaxed to 500 ppm [80,93]. Regarding materials, 13Cr stainless steel exhibits 20 times greater corrosion resistance than carbon steel in SO_2_-containing environments, despite a 25–30% cost increase [63]. Real-time monitoring using Fourier Transform Infrared Spectroscopy–Gas Chromatography (FTIR-GC) is recommended to dynamically adjust dehydration units and amine scrubber parameters, maintaining impurity concentrations within safe windows [86].

## 5. Future Perspectives

Based on a systematic review of the mechanisms by which impurities affect CO_2_ pipeline transportation, and considering current research gaps and technical challenges, future studies should focus on the following.

### 5.1. In-Depth Quantification and Prediction of Synergistic Effects of Impurities

While existing research has elucidated the mechanisms of individual impurities (e.g., H_2_O, O_2_, and SO_2_), there remains a lack of systematic and quantitative description of synergistic corrosion behaviors under the coexistence of multiple components (e.g., O_2_/SO_2_/NO_2_/H_2_S). Future work should develop multi-impurity reaction kinetics models to clarify critical thresholds for synergistic effects. Simultaneously, the regulatory mechanisms of impurities on phase separation should be analyzed to establish mapping relationships among impurity concentrations, phase behaviors, and corrosion rates.

### 5.2. Long-Term Dynamic Corrosion Behavior and Product Layer Stability

Current experimental durations are generally too short to simulate decades of pipeline service conditions. The long-term evolution of corrosion product layers (FeCO_3_/FeS)—particularly under high temperatures (>60 °C) or impurity perturbations (e.g., O_2_ > 500 ppm)—urgently needs investigation. Dense FeCO_3_ layers may transition into porous Fe_2_O_3_–FeSO4 mixed structures, losing protectiveness and initiating pitting. In situ characterization techniques should be developed to dynamically track critical conditions for film failure. Additionally, the accelerated stripping of corrosion products by erosion in dynamic flow fields requires the construction of cyclic flow experimental systems combined with slow strain rate tests to quantify risks of SCC and hydrogen-induced cracking (HIC).

### 5.3. Standardized Experimental Systems and Interdisciplinary Integration

Significant data variability severely restricts engineering applications. Methodological differences between static autoclave and dynamic flow loop tests lead to substantial discrepancies, primarily due to non-standardized aqueous phase contact modes (continuous phase vs. droplets) and impurity consumption. Unified protocols and dynamic parameter simulation standards must be established. Concurrently, interdisciplinary collaboration is urgently needed, including integrating materials science to develop low-cost corrosion-resistant alloys, leveraging fluid dynamics to model critical droplet sizes for predicting acid condensation locations, utilizing artificial intelligence to fuse large-scale corrosion data for long-term rate prediction models, and employing transfer learning to generalize behavioral patterns across steel grades.

### 5.4. Engineering-Oriented Optimization of Impurity Control

Existing engineering standards lack multi-field coupling foundations. Operational condition-adaptive regulation must be developed in order to dynamically adjust impurity thresholds under varying conditions, integrate temperature–pressure–impurity variables, and map critical corrosion rate boundaries into engineering charts. Simultaneously, material and mitigation strategies should be front-loaded, including embedding corrosion considerations during pipeline design, defining upper limits for free water and impurity tolerances, and developing closed-loop online monitoring and control systems to dynamically optimize dehydration units and amine scrubber operational parameters.

Breaking through the four major bottlenecks—synergistic impurity effects, long-term corrosion evolution, experimental standardization, and interdisciplinary integration—constitutes the core pathway to achieving safe transportation in CCUS sCO_2_ pipelines. Future efforts must synergistically advance fundamental mechanism research, engineering tool development, and standard system construction to provide scientific and technological support to the global carbon reduction infrastructure.

## 6. Conclusions

The impact of impurities on material integrity in sCO_2_ pipeline transportation is a nonlinear process triggered by H_2_O and dominated by the synergistic effects of multiple impurities. This review systematically dissected this complex mechanism, confirming that oxidizing impurities such as O_2_, SO_2_, and NO_2_ significantly accelerate corrosion by disrupting protective corrosion product layers or generating strong acids in situ. The strength of their synergistic effect far exceeds the simple sum of individual actions, constituting a major threat to pipeline safety. Consequently, the key to the successful engineering of protection lies in the strict combined control of water content and critical impurity concentrations, coupled with the selection of appropriate corrosion-resistant materials. Future work must focus on developing cross-scale theoretical models and innovative characterization techniques to provide scientific and technological support for the safe transportation of CO_2_ in the context of carbon neutrality.

## Figures and Tables

**Table 1:** Summary of corrosion mechanisms for different impurities.

Gas	Primary Impact Mechanism	Critical Concentration Thresholds and Corrosion Rate Data
H_2_O	Initiates the corrosion process, forms an acidic water film, causes minor uniform corrosion at low concentrations, and accelerates pitting and general corrosion at high concentrations.	<50 ppm initiates mild corrosion (hazardous with SO_2_), 100–1000 ppm initiates pitting (1.2 mm/y), >1000 ppm yields general corrosion (19 mm/y), and the corrosion rate of X65 steel increases sharply at water content > 1500 mg/L.
O_2_	Accelerates corrosion at low concentrations (disrupts FeCO_3_ film), may inhibit corrosion at high concentrations (forms dense oxides), and synergistically exacerbates corrosion with SO_2_.	1.5 ppm can increase the corrosion rate to >100 mm/y, 200 ppm raises the corrosion rate of X70 steel to 0.09 mm/y, the recommended concentration is <1000 ppm, and the corrosion rate reaches 20.47 mm/y when coexisting with 500 ppm SO_2_.
SO_2_	May inhibit corrosion at low concentrations, accelerates corrosion at high concentrations (generates strong acids), and synergistically forms H_2_SO4 with O_2_, significantly increasing the corrosion rate.	0.05% SO_2_ in liquid CO_2_ causes a corrosion rate of 2.4 mm/y, 500 ppm increases the corrosion rate of X70 steel to 1.10 mm/y, the recommended concentration is <100 ppm, and corrosion products on X65 steel become porous when coexisting with 3% O_2_.
H_2_S	Accelerates uniform corrosion at low concentrations, may mitigate corrosion via FeS film at medium–high concentrations, and corrosion products are regulated by the CO_2_/H_2_S pressure ratio.	<500 ppm accelerates corrosion, 0.0004 MPa H_2_S increases the corrosion rate of N80 steel to 4.61 mm/y, >0.4 MPa reduces it to 0.72 mm/y, and the recommended concentration is <200 ppm.
NO_2_	Generates HNO_3_, significantly reduces pH, accelerates uniform and localized corrosion, the effect is intensified at low temperatures, and the risk is amplified synergistically with O_2_/SO_2_.	100 ppm leads to a corrosion rate of 11.6 mm/y, the rate at 5 °C is 3–4 times higher than at 25 °C; <1.5 ppm is recommended, and the localized corrosion rate reaches 6.8 mm/y when coexisting with 1000 ppm O_2_.
N_2_/H_2_/CH_4_	Reduces water solubility, promotes free water separation, H_2_ may cause hydrogen embrittlement, CH_4_ alters phase behavior, and the total volumetric fraction should be <4%.	10% N_2_ reduces the water solubility by 30%, 4% H_2_ triples the corrosion rate, 20% CH_4_ decreases the water solubility, and corrosion intensifies with water separation when non-condensable gases >5%.

**Table 2:** Gas quality specifications for current European projects and related companies.

Medium Component	DYNAMIS Project		Northern Lights Project	Porthos Project	Ecofys Company	Pace CCS Company
	Content Limit Values		Content Limit Values (ppm/mol)	Content Limit Values (ppm/mol)	Content Limit Values	Content Limit Values (ppm/mol)
	Saline Aquifer Storage	CO_2_-EOR Project				
CO_2_	>95.5%		>99%	≥95%	>95%	≥95%
H_2_O	0.05%		≤30	≤70	<4%	50
Ar	<4%		--	≤4%	<4%	4%
N_2_			--		<4%	4%
H_2_			≤50		<4%	1%
CH_4_	<4%	<2%	--		<4%	4%
O_2_	<4%	0.01%~0.1%	≤10		<4%	10
CO	0.2%		≤100		--	0.2%
COS	--		--	≤20	--	5
H_2_S	0.02%		≤9		--	
SO_X_	0.01%		≤10		--	50
NO_X_	0.01%		≤10	≤5	--	50
Amines	--		≤20	≤1	--	100
C_2+_	--		--	1200	--	4.15%

## Data Availability

No new data were created or analyzed in this study.
